# Effect of Nebulized Morphine on Dyspnea of Mustard Gas-Exposed Patients: A Double-Blind Randomized Clinical Trial Study

**DOI:** 10.1155/2012/610921

**Published:** 2012-03-19

**Authors:** Majid Shohrati, Mostafa Ghanei, Asghar Amini Harandi, Soniya Foroghi, Ali Amini Harandi

**Affiliations:** ^1^Research Center of Chemical Injuries, Baqiyatallah Medical Sciences University, Mollasadra Street, P.O. Box 19945-546, Tehran, Iran; ^2^School of Medicine, Jahrom University of Medical Sciences, Jahrom 7414846199, Iran; ^3^Department of Neurology, Shahid Beheshti University of Medical Sciences and Health Services, Tehran 19839-63113, Iran

## Abstract

*Background*. Dyspnea is one of the main complaints in a group of COPD patients due to exposure to sulfur mustard (SM) and is refractory to conventional therapies. We designed this study to evaluate effectiveness of nebulized morphine in such patients. *Materials and Methods*. In a double-blind clinical trial study, 40 patients with documented history of exposure to SM were allocated to two groups: group 1 who received 1 mg morphine sulfate diluted by 4 cc normal saline 0.5% using nebulizer once daily for 5 days and group 2 serving as control who received normal saline as placebo. They were visited by pulmonologist 7 times per day to check symptoms and signs and adverse events. Different parameters including patient-scored peak expiratory flow using pick flow meter, visual analogue scale (VAS) for dyspnea, global quality of life and cough, and number of respiratory rate, night time awaking for dyspnea and cough have been assessed. *Results*. The scores of VAS for dyspnea, cough and quality of life and also respiratory rate, heart rate, and night time awaking due to dyspnea and night time awaking due to cough improved significantly after morphine nebulization without any major adverse events. Also pick expiratory flow has been improved significantly after nebulization in each day. *Conclusion*. Our results showed the clinical benefit of nebulized morphine on respiratory complaints of patients due to exposure to SM without significant side effects.

## 1. Introduction

Dyspnea is the most common complaint in patients with chronic obstructive pulmonary disease (COPD) [1]. As the disease progresses to severe COPD, dyspnea may often decrease quality of life and activities of daily living. Opioids have been utilized to suppress the sensation of dyspnea in the patient with chronic and progressive COPD as palliative therapy [2]. Systemic treatment with opioids has been reported to reduce dyspnea in some patients groups [3, 4], but adverse effects are common and limit their long-term use. Nebulized morphine is potentially attractive, since fewer side effects have been noted with inhaled morphine compared with injectable routes, and some controlled studies have suggested a beneficial effect on dyspnea [5–7]. This would offer an advantage if the relief of dyspnea could be relieved without considerable adverse effects [8].

In the patient with end-stage COPD, nebulized opioids may be considered as a potential treatment option [8]. Morphine may also decrease anxiety and diminish ventilatory response to hypoxia and hypercapnia [9]. Eaton and associates postulated that the mechanism of nebulized opioids in patients with lung disease, although not well understood, may be multifactorial. Depression of local opioid receptors in the lungs, spinal cord and central respiratory centers, and other systemic effects may all have a role [8, 10].

However, the evidences in the literature are still controversial and placebo-controlled studies to support nebulized morphine for the relief of dyspnea in patients with COPD are not sufficient [8].

Dyspnea is one of the main complaints in a group of COPD patients due to exposure to sulfur mustard (SM). Bronchiolitis obliterans (BO) is the main underlying long-term respiratory consequence in these patients [11, 12]. SM is one of the major potent chemical warfare agents. Unfortunately, Iraqis used sulfur mustard extremely against Iranian population during Iraq-Iran war in (1984–1988). As a result over the time about 100,000 Iranians were exposed to chemical warfare agents [13], and more than 30,000 survivals still suffer from respiratory complications [14]. The study was designed because of some reports asserting that inhaled opioids are able to relieve severe dyspnea from malignant or nonmalignant lung disease [5, 6]. Consequently, we designed this study for the first time to evaluate effectiveness of nebulized morphine in this group of patients comparing to the placebo group.

## 2. Materials and Methods

In a double-blind clinical trial study, 40 male patients with COPD due to exposure to SM were enrolled. Patients with history or abnormal laboratory tests indicating renal failure or cardiovascular diseases and history of adverse reaction to morphine were excluded.

They were allocated to two groups. Groups 1 who received morphine sulfate as inhaler and group 2 served as control who received placebo. In morphine group, patients consumed 1 mg morphine diluted by 4 cc normal saline 0.5% using nebulizer once daily for 5 days. The placebo group followed same instruction with just 5 cc normal saline 0.5%. They were visited by pulmonologist 7 times per day to check symptoms and signs and adverse events.

The physician and patients were kept blind about the consumed medication. We used PARI LC SPRINT nebulizer with same type and color in two groups. It is a nebulizer that can transport micro-aerocell with 2-3 microns in diameter to lower airways such bronchiole in adults. In assessing the potential benefit of inhaled morphine on dyspnea and related complaints in our patients, different parameters such as peak expiratory flow using pick flow meter, patient-scored visual analogue scale (VAS) for dyspnea (ranged from 0, that is, no dyspnea to 10, that is, worse dyspnea), cough (10-cm linear scale on which patients indicate the severity of their cough; 0 mm represents no cough and 10 cm the worst cough ever) were assessed. In addition, number of respiratory rate, night time awaking for dyspnea, and cough has been evaluated. Global quality of life was measured with a VAS: a horizontal line of 10 cm ranging from 0 (worst imaginable quality of life) to 10 (perfect quality of life). The study was performed according to the principles of the Declaration of Helsinki. Informed consent was obtained from all participating patients.

Statistical analysis was performed using SPSS version 16.0 software (SPSS Inc, Chicago, IL). The *t*-test and repeated measured analysis were used to compare data between and within groups. Values were presented as mean ± SD. A *P* value less than 0.05 was considered significant.

## 3. Results

Comparison of dyspnea, cough, and quality of life in two groups has been shown in Table 1. There were statistically significant differences between the morphine and the placebo group in VAS scores for dyspnea, cough, and quality of life. Also there was significant difference between two groups regarding mean difference of night time awaking due to dyspnea and night time awaking due to cough.  Table 2 shows mean of differences in respiratory rate, hear rate, night time awaking for dyspnea, and cough in two groups.

Results of 5-day mean pick flow meter of two groups over the time at 15th min, 30th min, 45th min, 1st h, 2nd h, 4th h, and 8th h after inhalation in each day are summarized in Table 3. *P* value for comparison of two groups is based on repeated measured analysis.

## 4. Discussion

The VAS scores for dyspnea, cough, and quality of life, and number of respiratory rate, heart rate, night time awaking due to dyspnea and night time awaking due to cough improved significantly after morphine nebulization without any major adverse events. Also pick expiratory flow rate has been improved significantly after nebulization in each day comparing to placebo. No considerable adverse reactions occurred in our study.

We used the VAS for evaluation of dyspnea in our series. The sensitivity of the VAS is enough to detect changes in breathlessness. Although, the Borg scale has greater reproducibility and is now widely used, the VAS has greater precision and sensitivity [15]. The specificity of the Borg scale depends on the instructions given to the subject [15, 16]. Furthermore we assessed global quality of life using VAS. The VAS is an instrument with good validity, excellent reliability, moderate distribution-based responsiveness, and good anchor-based responsiveness compared to multiitem questionnaires. Its use is recommended in clinical trials to assess global quality of life [17].

Opioids have been administered through a variety of routes to relieve dyspnea in patients with advanced COPD since the late 19th century. Morphine also was administered by inhalation to relieve dyspnea with least side effects that might have occurred when used via systemic routes [16]. The mechanisms of action of nebulized opioids are not clear. They may reduce the sensation of breathlessness primarily through a central effect on the brain. Also, it has been shown that low doses of morphine consumed directly to the lung via nebulizer are effective in some patients. Three main opioid receptors have been identified in the respiratory tract: *μ* (MOR), *δ* (DOR), and *k* (KOR), which mediate the effects of the 3 primary families of endogenous opioids (endorphins, enkephalins, and dynorphins, resp.) as well as exogenous opioids such as morphine and codeine [18]. In addition, the lungs also may contain a novel opioid receptor [19, 20]. Among them the *k* receptor is the predominant opioid receptor in the lung [21]. An additional suggested mechanism for the therapeutic effects of inhaled morphine might be the inhibition of pulmonary-irritant receptors [22]. Opioids depress the release of proinflammatory “substance P,” which may help to decrease local inflammation [18]. We also suppose the possible role of opioid to act on neurogenic inflammation process as another involved mechanism for palliation of respiratory symptoms. Therapeutic aerosols have a wide range of particle sizes and shapes. It was concluded that in particle size of 2.3 to 3 *μ*m, a solution of morphine was more likely to place in the peripheral airways during tidal breathing than particle size in 4.9 *μ*m, which tends to cause impaction in the central airways [23]. In our study a nebulizer that delivers a small particle size (mass median diameter 3 *μ*m) was used for reaching better to bronchioles where there is location of main pathology of BO.

The effect of nebulized morphine on dyspnea has been remained controversial until now and different studies reported mixed results [8]. Most studies using doses of 1–25 mg have not shown considerable benefit from inhaled opioids on dyspnea [24–32], but other ones showed a beneficial effect [33, 34]. Young et al. reported an increase in exercise endurance in COPD patients after a low 5 mg dose of nebulized morphine [35]. In a small trial, a single dose of morphine-6-glucuronide was administered to nine breathless patients with cancer at dose levels of 5 mg, 10 mg, and 20 mg. All patients reported improvement in dyspnea by VAS and Borg scale with no apparent differences among doses [36, 37]. In a study of 18 patients with COPD, Sato et al. demonstrated that 4 mg nebulized morphine-6-glucuronide increased exercise endurance [28]. Also, there have been several reports concerning the effectiveness of nebulized morphine in relieving cancer-related dyspnea [5, 9, 38].

On the other hand, there are some studies against effectiveness of morphine in respiratory complaints. Foral and colleagues reviewed seven studies on patients with a variety of cardiorespiratory disorders who received nebulized morphine. In five of studies on COPD, one study evaluated a mix of pulmonary and nonpulmonary patients, and another study was as a small cohort of patients with interstitial lung disease (ILD). The authors concluded that the evidence did not support the use of nebulized morphine for the relief of dyspnea or in the improvement of exercise tolerance in patients with COPD or ILD. They recognized that differences in dose, administration schedule, and inconsistent use of oxygen and bronchodilators could have contributed to the variability in results. However, reported side effects were few and mild. Furthermore, it was suggested that the underlying disease for which the aerosol is delivered may influence the deposition of particles [16].

In our study patients were not on oxygen therapy and all were receive long-acting bronchodilator twice per day during the 5-day study period. Our result showed desirable effectiveness of once daily low dose nebulized morphine on dyspnea and related consequences in SM exposed BO. It is very interesting because most trials have investigated the effects of aerosolized opioids on dyspnea and exercise tolerance in patients with stable chronic cardiopulmonary disease and found no effect [39]. However, some point should be noted in this setting. There is no evidence of emphysema as a consequence of disrupted lung parenchyma in our patients [11]. Furthermore, they have not had other risk factors that are seen in common cases of COPD and BO. These should be considered as appreciate reasons for effectiveness of nebulized opioid in our setting. Of note, there is an irreversible air flow limitation in such chemical injured that has not respond to routine therapies.

Although, insufficient well-designed studies are one of the most important reasons for reluctance to prescription of opioids, there are other limitations as well. They include fear of respiratory depression and addiction in both physicians and patients groups [40, 41]. There was no significant increasing in adverse effects in most studies on nebulized morphine. Also, the long-term prescription of opioid as a palliative therapy for respiratory symptoms may lead to temporary physical dependence that should not be confused with addiction [42]. However, nebulization has a number of advantages. First, nebulized opiates may be better tolerated at higher doses than systemic administration. It may be devoid of serious side effects because its bioavailability is very small [43]. Second, relief is likely to be more rapid than by oral intake. Relief of dyspnea has been reported to occur shortly after or within 10–15 min of application of nebulization treatment [40]. Third, patients can manage their dyspnea by themselves easily, when dyspnea occurs or worsens. These advantages suggest the possibility of the use of nebulized morphine for patients at home as a rescue treatment during dyspnea attack or prophylactically before daily activities [44].

It appears that nebulized opium benefit lasts for a few hours and an increase in the dose up to 80 mg and in frequency up to every 2 h is tolerable [29]. Interestingly, our results revealed that its benefit remained up to 8 hours. In this study, nebulized doses of morphine lower than those previously reported were administered. The effect of nebulized morphine is unlikely to be caused by systemic absorption of the nebulized dose. Some reasons for this fact are as follows. The bioavailability of nebulized morphine has been reported 5.5% (range 5–35%) given that much of the drug is deposited in the delivery systems [45–47], but systemic doses required affecting dyspnea range from 5 to 50 mg. Furthermore, the mean delay to obtain a peak serum concentration following nebulization is 45 min [45]. The maximum serum morphine concentration was achieved by 45 min and was approximately 6 times lower than with intramuscular administration [39]. We found that least dose, that is, 1 mg nebulized morphine shows its effect on dyspnea just after 15 min; the time before a peak serum concentration supports central pulmonary mechanism of action rather than systemic effects. Of note in cancer patients who suffer from intractable dyspnea, relatively small amounts of inhaled opioids appear to improve breathing comfort, despite the fact that these patients already are receiving high levels of parenteral opioids for pain management [5, 9, 29, 39, 44, 48].

This study has some limitations. The plasma concentrations of morphine have not been measured. Also the time of followup was not sufficient. Assessment of severity using spirometry was not feasible, thus we could not measure volumes and just flow was measured. Although flow is dependent to volume, it could be influenced by other variables like respiratory muscles work. Its effective use in clinical practice needs further examination. How long its effectiveness sustains, how many times it can be used safely, and what the limiting factor of this treatment is are very important remaining questions. However, practical prescription of nebulized morphine in this field needs more evaluation with larger sample size and longer followsup period. Complementary study with crossover method is appreciated.

## 5. Conclusion

Our study on COPD patients resulting from SM exposure revealed that nebulized morphine is effective for reducing dyspnea and related complaints and can be used safely in parallel with current therapies in this setting.

## Figures and Tables

**Table 1:** Comparison of dyspnea, cough, and quality of life within and between two groups.

	Group	Before	Day 1	Day 2	Day 3	Day 4	Day 5	*P* value
Dyspnea	Morphine	6.9 ± 1.1	6.1 ± 1.1	5.7 ± 0.9	5.3 ± 1.0	5.2 ± 1.1	5.1 ± 1.0	<0.001
	Placebo	7.1 ± 1.5	7.4 ± 1.5	7.3 ± 1.5	7.2 ± 1.4	7.2 ± 1.4	7.2 ± 1.4	

Cough	Morphine	5.8 ± 1.8	5.0 ± 1.8	4.6 ± 1.6	4.2 ± 1.7	4.1 ± 1.8	4.1 ± 1.7	<0.001
	Placebo	5.5 ± 1.9	5.6 ± 1.9	5.5 ± 1.9	5.5 ± 1.9	5.5 ± 1.9	5.5 ± 1.9	

Quality of life	Morphine	4.5 ± 1.3	4.7 ± 1.1	4.7 ± 1.7	4.7 ± 1.7	4.8 ± 1.1	5.0 ± 1.2	0.03
	Placebo	3.4 ± 1.5	3.4 ± 1.4	3.5 ± 1.5	3.5 ± 1.5	3.5 ± 1.5	3.5 ± 1.5	

**Table 2:** Mean of differences in respiratory rate, hear rate, night time awaking for dyspnea, and cough in two groups.

	Morphine	Placebo	*P* value
Respiratory rate	1.5 ± 1.1	0.1 ± 0.3	<0.001
Heart rate	1.7 ± 1.6	0.45 ± 0.6	0.004
Night time awaking for dyspnea	1.0 ± 0.6	0.5 ± 0.6	<0.001
Night time awaking for cough	1.2 ± 0.7	0.5 ± 0.2	<0.001

**Table 3:** Pick flow meter over the time in total 5 days after inhalation in two groups.

	Baseline	15 min	30 min	45 min	1 h	2 h	4 h	8 h	*P* value
Morphine	66.7 ± 20.4	84.75 ± 48.6	104.75 ± 53.5	97.7 ± 52.7	96.25 ± 49.6	89.2 ± 51.4	89.0 ± 51.6	84.2 ± 47.6	0.017
Placebo	56.7 ± 23.1	63.2 ± 21.9	75.0 ± 12.7	73.5 ± 12.8	64.7 ± 26.0	71.0 ± 16.1	68.5 ± 19.1	67.1 ± 18.2	
